# The effect of human autonomy and robot work pace on perceived workload in human-robot collaborative assembly work

**DOI:** 10.3389/frobt.2023.1244656

**Published:** 2023-11-03

**Authors:** Wietse van Dijk, Saskia J. Baltrusch, Ezra Dessers, Michiel P. de Looze

**Affiliations:** ^1^ Healthy Living, TNO, Leiden, Netherlands; ^2^ HIVA, KU Leuven, Leuven, Belgium

**Keywords:** cobot, perceived workload, industrial assembly work, autonomy, work pace, job quality

## Abstract

Collaborative robots (in short: cobots) have the potential to assist workers with physically or cognitive demanding tasks. However, it is crucial to recognize that such assistance can have both positive and negative effects on job quality. A key aspect of human-robot collaboration is the interdependence between human and robotic tasks. This interdependence influences the autonomy of the operator and can impact the work pace, potentially leading to a situation where the human’s work pace becomes reliant on that of the robot. Given that autonomy and work pace are essential determinants of job quality, design decisions concerning these factors can greatly influence the overall success of a robot implementation. The impact of autonomy and work pace was systematically examined through an experimental study conducted in an industrial assembly task. 20 participants engaged in collaborative work with a robot under three conditions: human lead (HL), fast-paced robot lead (FRL), and slow-paced robot lead (SRL). Perceived workload was used as a proxy for job quality. To assess the perceived workload associated with each condition was assessed with the NASA Task Load Index (TLX). Specifically, the study aimed to evaluate the role of human autonomy by comparing the perceived workload between HL and FRL conditions, as well as the influence of robot pace by comparing SRL and FRL conditions. The findings revealed a significant correlation between a higher level of human autonomy and a lower perceived workload. Furthermore, a decrease in robot pace was observed to result in a reduction of two specific factors measuring perceived workload, namely cognitive and temporal demand. These results suggest that interventions aimed at increasing human autonomy and appropriately adjusting the robot’s work pace can serve as effective measures for optimizing the perceived workload in collaborative scenarios.

## 1 Introduction

The fourth industrial revolution, conceptualized in Industry 4.0, has led to the introduction of various new technologies that digitize, connect, and automate procedures. Despite this ever-increasing level of automation, human involvement is still crucial due totheir adaptability, dexterity, and cognitive abilities. To make optimal use of the strengths of humans within a highly automated environment new solutions are needed. The development of the collaborative robot or cobot, allowed humans and robots to work closely together as a flexible and efficient team (Lenz et al., 2008). A key characteristic of human-robot collaboration is the interdependency between human and robot actions (Hoc, 2000).

The benefit of human-robot collaboration (HRC) is the possibility to make optimal use of human and robot strengths and mitigate weaknesses. HRC can improve efficiency through concurrent motion of robots and humans (Lasota and Shah, 2015). The potential benefit for humans in HRC is that it can reduce physical and cognitive workload (Singh et al., 2013; Cherubini et al., 2016). On the other hand, working in collaboration with a robot can also have negative effects for humans. To facilitate human robot collaboration, more focus on the human is needed, instead of automation alone (Kolbeinsson, Lagerstedt, and Lindblom, 2019).

To measure and access the quality of jobs the OECD developed the framework for job quality (Cazes, Hijzen, and Saint-Martin, 2015). Within this framework, the quality of the working environment is the dimension that deals with the non-economic aspects of the work. A good working environment balances job demands and job resources. This balance originated from the job-demand-control model (Karasek, 1979). According to this model operator wellbeing depends on the balance between the level of job demands, and the level of control the operator has to cope with these demands. The introduction of robots in the workplace alters both demands and control. The demands change when tasks are reallocated between the human and the robot, or the robot changes the working pace. The level of control changes when the robot takes over decision making tasks from the human. In the design of HRC applications, numerous task distributions and robot work paces can be considered, offering an opportunity to optimize the HRC implementation and enhance working conditions. In this research, we focus on the robot work pace and the human autonomy, i.e., the level of control an operator has to select and initiate actions.

Human autonomy in HRC is conceptualized in the levels of automation that describe ten levels between fully manual and fully automated behavior (Parasuraman, Sheridan, and Wickens, 2000). According to this study, an automation level should be chosen that optimizes performance. A metastudy Onnasch et al. (2014) shows a preference to increase automaton until a tipping point is reached where the unwanted effects from mistakes overtake. HRC studies by Gombolay et al. (2015) and Schulz et al. (2017) have shown that humans prefer working with a robot with a relative high level of automation. This seems to suggest that operators are willing to sacrifice autonomy if there is a considerable advantage in demand. The opposite has been argued by Weiss et al. (2011) who stated that operators might perceive a negative change in their working conditions when part of the control and task load is taken over by the robot. In line with this Pollak et al. (2020) found that manual control over the robot improved the wellbeing of the operator.

The effect of pace and synchronization of human and robot actions has been captured in the concept of fluency for which a set of subjective and objective metrics is available. The objective metrics include the relative portion of functional and non-functional delays of the human and robot, and the amount of parallel work (Hoffman, 2019). Fluency is generally improved by minimizing delays, especially for the worker. This promotes a fast-paced robot that finishes tasks early in anticipation of human tasks. However, two studies revealed that a high moving speed of the robot leads to high cognitive workload, significantly increasing fear, surprise and discomfort (Arai, Kato, and Fujita, 2010; Fujita, Kato, and Tamio, 2010). These results might also be explained in part by the fact that faster moving robots increased the sense of time pressure.

The aforementioned studies seem to make conflicting statements about the role of human autonomy and time pressure. There are several explanations for this. First, the change in human autonomy or work pace is paired with other factors that have influenced the outcomes. For example, time pressure and robot speed might be influenced at the same time (Arai, Kato, and Fujita, 2010; Fujita, Kato, and Tamio, 2010), or the change in autonomy also entails a change in the task load (Fournier et al., 2022). Second, many studies are intended as a proof of concept and only involve a small (<10) number of subjects (Baltrusch et al., 2021).

To properly study the effects of time-pressure and autonomy, the conditions should be kept uniform in terms of task load. Such a standardization might also benefit industrial applications. Many industrial processes, such as assembly work, are characterized by repetitive tasks that must be completed in a prescribed cycle time (Cohen et al., 2022). This cycle time is linked to the task at hand and also to other tasks in the process and customer demand. HRC solutions that improve workload at the expense of cycle time are likely to be rejected in practice.

This study aims to identify the effect of human autonomy and robot work pace in the context of industrial assembly work. The following research questions are formulated:

1. What is the impact of increasing human autonomy on the perceived workload of industrial operators?
2. What is the impact of slower robot pacing on the perceived workload of industrial operators?

We have set up an experiment to answer the stated research questions. In the experiment, participants worked together with a robot on a manual assembly task. The task simulates a typical industrial assembly task. During the experiment the level of human autonomy and the robot work pace of the robot could be controlled such that multiple conditions were created that provided the data to answer the research questions.

## 2 Methods

### 2.1 Participants

In this study 20 participants were included (15 men and 5 women, 39 std 14 years old). Participants were recruited via flyers and personal contacts at the BIC Manufacturing Campus, Eindhoven, Netherlands and from the research groups at TNO and HIVA KU Leuven. As such, a diverse group of participants was found, 4 participants had secondary education or an associate degree, 4 had a bachelor’s degree, and 12 had a master’s degree or higher. 6 participants were students, 14 had a job. This research complied with the tenets of the Declaration of Helsinki and was approved by the Institutional Review Board at TNO, Leiden, Netherlands (application 2020-063). Informed consent was obtained from each participant.

### 2.2 Experimental set-up

**FIGURE 1:**
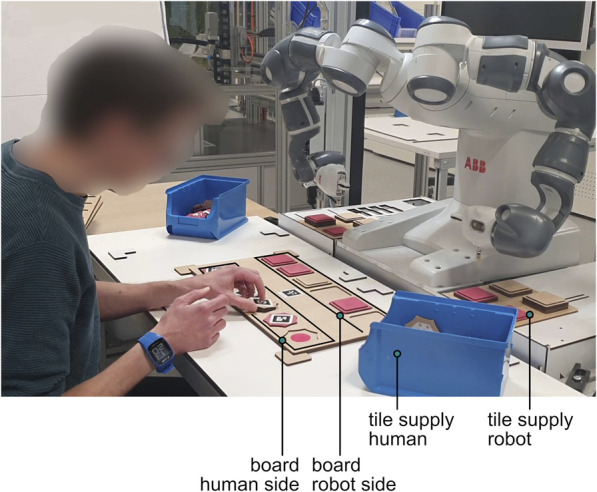
Experimental set-up of the manual assembly task.

The participant and the robot performed collaborative manual assembly tasks in a shared workspace (Figure 1).

**FIGURE 2:**
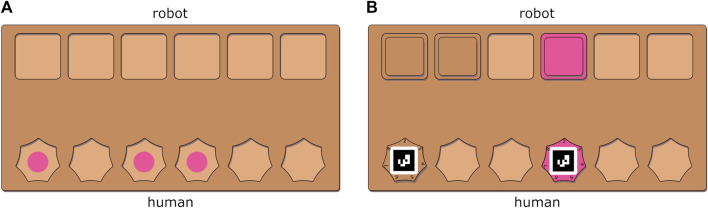
Tile boards for the collaborative tasks. **(A)**: tile board for the HL condition without tiles. Note that for the HL conditions the dots in the middle in the bottom rows indicate the color of the tile that needs to be placed. **(B)**: tile board for the FRL and SRL conditions with 3 robot tiles and 2 human tiles.

**FIGURE 3:**
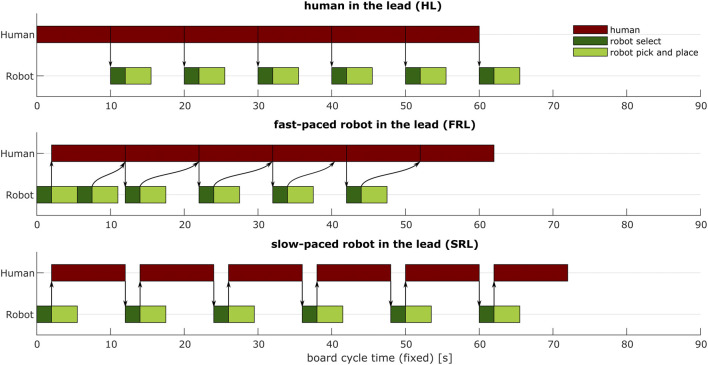
Timeline of the three conditions (HL, FRL, SRL). Task times are indicative for flawless task execution without delays or mistakes. The board cycle time, the time to finish one board, is fixed to 90 s. Arrows denote a dependency between an ending and a starting task. Note that when a human task is dependent on a robot task, the human can start as soon as the robot has selected a tile, and the human does not have to wait until the robot has completed the task.

The collaborative task comprised placing pink and brown tiles on a board. Each board had two rows with six slots and ten of these boards were made per condition. The human and the robot each filled a different row with tiles (Figure 2). The participant and the robot were instructed to place the tiles such that opposing tiles had the same color. Each participant collaborated with the robot in three different conditions. For each condition the way the tiles had to be placed on the board was different: human in the lead (HL), slow-paced robot in the lead (SRL), fast-paced robot in the lead (FRL). The actor that was in the lead, determined the placement order of the tiles and triggering the actions of the other actor (human or robot). The HL *versus* the FRL condition tests the effect of human autonomy (high *versus* low human autonomy). The SRL *versus* the FRL condition tests the effect of robot work pace. The robot work pace was changed by altering the onset of the robot movement. The robot speed was constant across conditions. The different conditions are visualized in Figure 3. A video of the conditions is available as Supplementary Material.

#### 2.2.1 Human in the lead (HL)

The participants initiated the task, by selecting a tile from their supply and placing it in one of the slots on the board (Figure 2A). In this condition the slots were marked pink or brown and the participant was instructed to place tiles in the slots with matching colors. Each board had a different color pattern to prevent that a participant learned a sequence. The participant could select its own placement pattern, i.e., the order in which the slots were filled. When the participant placed a tile, the robot picked a tile of the same color and placed it in the matching slot on the opposite side of the board. The human did not have to wait for the robot to continue to the next tile, so the participant and the robot worked in parallel.

#### 2.2.2 Slow-paced robot in the lead (SRL)

The robot initiated the task, by selecting a tile from its supply and placing this tile in one of the slots on the board (Figure 2B). Then participants had to pick a tile of the same color and placed it in the opposing slot on the board. The robot waited until the participant had placed a tile before placing its next tile. The participant and the robot worked serially on the task. The robot had a limited set of predefined placement patterns, to prevent that a participant learned a sequence.

#### 2.2.3 Fast-paced robot in the lead (FRL)

This condition was the same as the SRL condition with one exception. In the FRL condition the robot was allowed to work one tile ahead of the human so the participant and the robot worked in parallel without waiting times between human and robot tasks.

#### 2.2.4 Cognitive task

**FIGURE 4:**
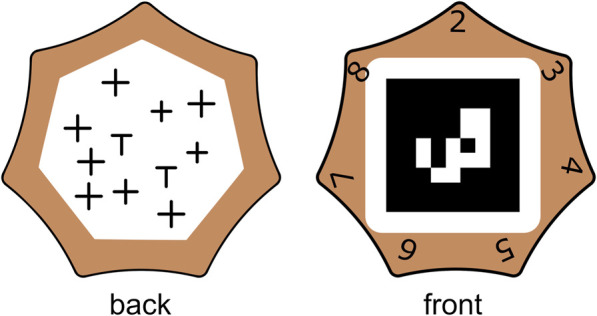
Back and front view of a tile that was placed by the human. The correct answer (2) is on top.

To assure, for each condition, that the task time of the robot was shorter than the task time of the human, the participants had to perform a small cognitive task before placing the tile. The participant had to count the number of “T”-signs in an arrangement of “T” and “+”-signs on the back of each tile (Figure 4). Each tile had between 2 and 8 “T”-signs on the back.

The front side listed multiple possible answers (2-8). The participant had to place the tile with the correct answer on top the board. To assure that the participant placed all the tiles correctly. The tiles had an unnoticeable small asymmetry, such that the tiles only fitted in the slots when the correct answer was on top. When the participant noticed the tile did not fit, the participant had to correct the counting error before proceeding with the next tile. This approach effectively mitigated the possibility of errors at task completion.

#### 2.2.5 Robotic setup

The robotic setup consisted out of a dual armed YuMi cobot (IRB 14000, ABB, Zürich, Switzerland) and an auxiliary camera (Logitech C920 HD Pro Webcam). The board and robot-tiles supply had fixed positions and the robot was programmed to place tiles from its tile supply to the board using its build in suction cups. The position and color of the tiles that were placed on the board by the participant were detected by the camera that tracked the square AR markers that were put on the tiles and board. The detection of a new placement triggered the robot actions. The synchronization of tasks performed with custom scheduling software (Pupa, Van Dijk, and Secchi, 2021). The robot motions were programmed in ABB RAPID software. The markers detection was programmed in ROS (kinetic) using the ar_track_alvar package.

### 2.3 Experimental procedure

Before the start of the measurement participants were requested to fill in a questionnaire on personal characteristics, gender, age, highest completed level of education and type of employment. After a short explanation of the experimental set-up, each participant started with a try-out where each condition was tested for a brief period. During this try-out the participants could get familiarized with the robot and the task. Subsequently, the participant performed the manual assembly task in the three different collaboration conditions (Figure 2). Each condition consisted of the assembly of ten boards in a row. The assembly of a board consisted of placing the tiles, removing the tiles placed by the robot, putting away the current board, and placing a new board on the table. The board cycle time, i.e., the time a participant had to finish one board, was fixed at 90 s. The board cycle time was established during a pilot and allowed the subjects to work at a comfortable pace. If the participant finished early, the participant waited until the 90 s were passed. If the participant was not ready in the allotted time the participant was allowed to finish the task before starting the next cycle. After each condition with ten boards, participants filled a questionnaire for assessing perceived workload and perceived performance. The sequence of conditions was systematically varied to prevent order effects.

### 2.4 Measurements

#### 2.4.1 Perceived workload

The NASA Task Load Index (TLX) (Hart and Staveland, 1988) was used to score the perceived workload on 6 scales: cognitive demand, physical demand, temporal demand, effort, frustration and perceived performance. A copy of the questionnaire in Dutch and English is available as Supplementary Material. Since objective performance was fixed through the board cycle time across conditions, the perceived performance score will serve an indicator of whether subjects perceived their performance as similar across conditions. The other factors are indicators for the change in perceived workload.

#### 2.4.2 Objective task performance

The board cycle time was kept constant (see Section Experimental set-up), while the tile cycle time had the potential to vary. To ensure that large variations in tile cycle time were not present across conditions, the tile cycle time was recoded. The tile cycle time was recorded as the time between placing two tiles by the participant. Any waiting time for the participant due to the robot was included in the cycle time. For each participant and condition the median cycle time was calculated. The placement of the first tile was discarded because it often had some irregularities in the recording and in the HL condition involved a vocal “go” from the experiment conductor which did not exactly line up with the start of the recording.

#### 2.4.3 Statistics

To test for statistical differences in collaboration conditions, the scale values of the TLX were compared between the conditions, using the non-parametric Wilcoxon test. The results will report the comparison between HL vs. FRL and SRL vs. FRL that relate to respectively research questions one and two. *p*-values below 0.05 were marked as statistically significant. Results will report the relevant findings.

## 3 Results

### 3.1 Perceived workload

**FIGURE 5:**
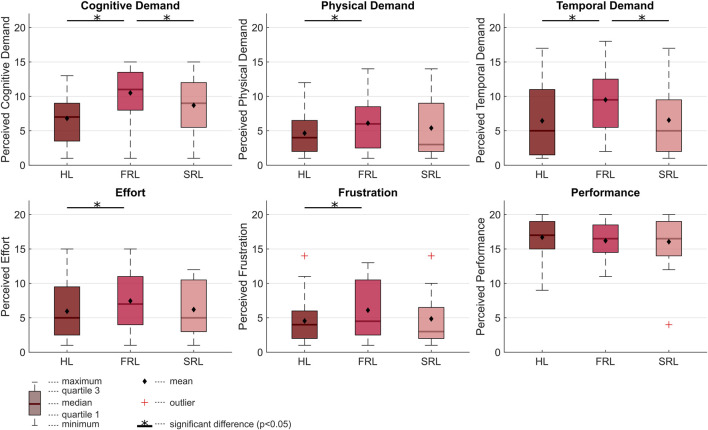
Perceived workload in the three collaboration conditions: Human in the lead (HL), Slow-paced robot in the lead (SRL) and Fast-paced robot in the lead (FRL).

The perceived workload (cognitive demand, physical demand, temporal demand, effort, frustration) and perceived performance are shown in Figure 5, a full report on the outcomes of the statistical tests is available as Supplementary Material.

Increased human autonomy (HL vs. FRL) led to a significant decrease in all perceived workload factors (cognitive demand *p* < 0.001, physical demand *p* = 0.011, temporal demand, *p* = 0.007, effort *p* = 0.03, frustration *p* = 0.032). Decreased robot work pace (SRL vs. FRL) led to a significant decrease in cognitive demand (*p* = 0.026) and temporal demand (*p* = 0.008). The same trend was observed in the other perceived workload factors, physical demand, effort, and frustration, but without significant differences. The difference in perceived performance between all collaboration conditions was small and not significant.

### 3.2 Objective performance

**FIGURE 6:**
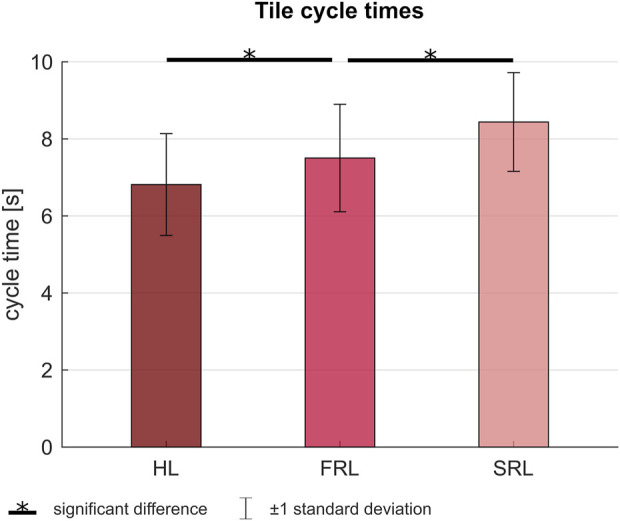
Average tile cycle times.

The tile cycle time, the time between two tiles placed by the human is shown in Figure 6.

Increased human autonomy (HL vs. FRL) led to a 9.2% decrease in tile cycle time (6.8 s vs. 7.5 s). Decreased robot work pace (SRL vs. FRL) led to an 12.5% increase in tile cycle time (8.5 s vs. 7.5 s). These differences were significant.

## 4 Discussion

### 4.1 Experimental validity and limitations

The experiment aimed to keep performance constant across conditions by fixing the board cycle time. Despite this, small (<15%) but significant differences in the tile cycle times were observed, which affected the waiting time between boards. However, these differences in tile cycle time did not lead to significant differences in perceived performance among participants. Furthermore, the increase in perceived workload was not directly associated with an increase in tile cycle time, as observed in the HL vs. FRL comparison but not in the SRL vs. FRL comparison. The tile cycle time is therefore not considered as a primary indicator for perceived workload. Still, the tile cycle time differences will be considered when interpreting the other results.

After the experiment the participants were asked if they were able to systematically improve their task execution with something they discovered in the experiment. First, they were asked after the experiment whether they recognized the predefined placement patterns of the robot, which was not the case. Secondly, participants were asked whether they discovered strategies that let them work more efficient, e.g., counting strategies for the cognitive tasks. Participants reported a wide range of strategies. However, they did not experience one strategy to be much more efficient that other strategies. Therefore, it is unlikely that placement patterns of the robot or the development of task strategies led to instantaneous changes in performance and influenced the outcomes of the experiment.

There are limitations to consider in this study. Firstly, our experiment focused solely on a single task resembling an industrial assembly task. The perceived workload, measured using the TLX, served as a proxy for job quality, but this relationship is non-linear. Extreme workload levels, either too low or too high, can lead to performance decline, following a U-model (Young and Stanton, 2002). Therefore, the findings should be interpreted within the context of industrial assembly work and may not be generalizable to other tasks, such as monitoring or high-load tasks. Also, factors of the TLX tend to correlate (Hart, 2006). This study reports the outcomes on all six factors. Due to the correlation between factors, it is difficult to isolate the effects on different types of perceived demands which might also be reflected in the results. For example, the perceived physical demand changed along with the other factors even though the real physical demand did not change.

Another limitation is that a fixed delay was chosen between human tasks, ensuring all participants experienced the same waiting time. This delay was effectively zero in FRL and HL conditions and small in the SRL condition. Consequently, participants were unable to adjust the waiting time by working at a faster or slower pace. In situations where the waiting time between tasks is dependent on the working speed of the human, the relation between working pace and perceived workload may differ.

Furthermore, the experiment involved modifying human autonomy by allowing participants to initiate tasks and select the execution order, which slightly altered their task load. The primary components of task load were manual handling and cognitive counting. However, minor variations in task load existed across the conditions. These limitations emphasize the need for caution when extrapolating the results, particularly to other types of work.

### 4.2 Human autonomy (HL vs. FRL)

The study results indicate that, in the assembly task, increased human autonomy reduces perceived workload. The increase in human autonomy (HL vs. FRL) was achieved by letting the human start the tile placement sequence and letting the human select the order in which the tiles were placed instead of the robot. This relatively small change in human autonomy was sufficient to lead to a significant decrease in the selected five perceived workload factors. This is in line with the findings of Pollak et al. (2020) that promotes manual control over the robot. This finding is also in line with the Karasek’s job-demand-control model (Karasek, 1979; De Spiegelaere et al., 2015). According to the model, when the operator has the job control (i.e., autonomy to initiate a new cycle) to tackle a matching job demand (i.e., work pace) the perceived workload will be lower. Thus, it should be noted that leaving a task for the operator to perform does not automatically increase perceived workload. On the contrary, take away a task which helps the operator control the job demands, and the perceived workload will likely increase. In contrast, the result of the present study conflicts the findings of (Gombolay et al., 2015; Schulz, Kratzer, and Toussaint, 2017) that promote automation. A difference between this study and Schulz et al. (2017) and Gombolay et al. (2015) is that these studies is that a pro-active involvement of the cobot resulted in clear task performance advantages, such as reduced execution time or less re-scheduling.

It was also observed that the tile cycle time in the FRL condition was slightly higher than in HL condition. This was not caused by increased waiting time of the human since the robot always worked ahead of the human (Figure 3). The change in autonomy also entailed a change in dependency between the robot and the human tasks. In the HL condition there was no direct dependency of the human tasks on the robot tasks, i.e., the human could work without noticing the result of the robot’s task. In the FRL condition, the human did have to watch the outcome of the robotic tasks, i.e., observe the color and the location of the tile placed by the robot. The dependency on robot tasks in the FRL condition might have caused that the participant felt an increased need to actively follow the robot’s actions. It could have also been caused by the fact that since the robot took the initiative, it was perceived as less predictable. The predictability of robot motions has been positively associated with trust and perceived safety (Dragan et al., 2015). Both factors might have led to an increase in attention to the robot’s actions which might have caused the increased cognitive and temporal demand in the FRL condition.

### 4.3 Robot work pace (SRL vs. FRL)

This study indicates that decreased robot working pace reduces workload. In the SRL condition, participants experienced a fixed delay between their tasks. They had to wait with picking a new tile until the robot selected a tile color by picking a new tile from the supply. This contributed to the observed increase in the tile cycle time. In the FRL condition the new tile from the robot was already on the table since the robot worked one step ahead. This change led only to a significant change in two factors, cognitive and temporal demand, and is thus less prominent as the human autonomy related effect.

The finding that workload indicators are lower in the SRL condition competes with the findings of Hoffman (2019) who found positive associations between objective fluency (i.e., minimizing delays) and factors such as trust and bonding which favor the FRL condition. However, if reducing workload is the goal, it can be achieved by decreasing the robot pace, which aligns with the SRL condition. Other studies (Arai, Kato, and Fujita, 2010; Fujita, Kato, and Tamio, 2010) have shown that faster moving robots increase mental strain. It must be noted that in their studies the moving speed of the robot was increased. This study changed pace, i.e., the timing of the onset of robot actions, alone and the robot moving speed was constant. Changing the moving speed of the robot might have a separate effect on perceived workload.

## 5 Conclusion

The study demonstrates that human-robot collaboration (HRC) in industrial settings creates interdependence between humans and robots, which can impact job quality. Based on The OECD Job Quality Framework and Karasek’s job-demand-control model (1979), human autonomy and robot work pace were selected as key factors that might affect the perceived workload. The experiment manipulated these two factors across three conditions.

Increasing human autonomy by assigning decision-making tasks to humans resulted in a decrease in perceived workload, even for small decision-making tasks typical in industrial assembly. This finding aligns with the notion that higher levels of human autonomy which match corresponding job demands contribute to improved job quality.

Lowering the work pace of the robot, such that it creates small waiting times between tasks for the human, led to a reduction in perceived workload. This finding supports previous research suggesting that a high working speed of the robot can increase mental strain. Interestingly, this finding contradicts the fluency principle, which emphasizes minimizing waiting times for both humans and robots.

These findings have practical implications for various industrial HRC processes that involve a sequence of human and robot tasks. The level of human autonomy can be adjusted by determining task initiation and execution order responsibilities between humans and robots. Similarly, the robot’s pacing can be modified by altering the timing of its actions. Importantly, these changes can be implemented independently of the primary task distribution between humans and robots, without significant consequences for productivity. Based on the study’s results, two design guidelines are proposed to optimize HRC applications:

• Encourage a design that allows operators some freedom to initiate tasks and choose the execution order.
• The work pace of a robot can be optimized by balancing fluency, cognitive demands, and temporal demands (time pressure). Lowering the robot pace can be an effective strategy to reduce cognitive and temporal demands.

By following these design guidelines, industrial HRC processes can be optimized to enhance working conditions, improve job quality, and mitigate workload-related challenges.

## Data Availability

The datasets presented in this article are not readily available because the data involves human subject data. This data should only reported on group level. Requests to access the datasets should be directed to wietse.vandijk@tno.nl.
