# Supplementary material for: The effect of human autonomy and robot work pace on perceived workload in human-robot collaborative assembly work
Source: Front Robot AI. 2023 Nov 3;10:1244656. doi: 10.3389/frobt.2023.1244656 (PMC10655125; doi:10.3389/frobt.2023.1244656)
Supplement: Supplementary file 1 [file DataSheet1.pdf]

Supplementary Material

The Effect of Human Autonomy and Robot Work Pace on Perceived Workload in Human-Robot Collaborative Assembly Work

Wietse van Dijk*, Saskia J Baltrusch, Ezra Dessers, Michiel P de Looze

*** Correspondence:** wietse.vandijk@tno.nl

Statistical tests

Table 1 - Table 6 show the outcomes of the Wilcoxon Signed Ranks Tests. The comparison Fast Robot Lead (FRL) with Human Lead (HL) tests the effect of human autonomy. The comparison FRL with Slow Robot Lead (SRL) tests the effect of robot work pace.

Table 1: Test Statistics Cognitive

	SRL-HL	FRL-HL	FRL-SRL
Z	-2,054[a]	-3,519[a]	-2,230[a]
Asymp. Sig. (2-tailed)	,040	<,001	,026
a. Based on negative ranks.			

Table 2: Test Statistics Physical

	SRL-HL	FRL-HL	FRL-SRL
Z	-1,610[a]	-2,546[a]	-,526[a]
Asymp. Sig. (2-tailed)	,107	,011	,599
a. Based on negative ranks.			

Table 3: Test Statistics Temporal

	SRL-HL	FRL-HL	FRL-SRL
Z	-,026[a]	-2,676[b]	-2,667[b]
Asymp. Sig. (2-tailed)	,979	,007	,008
a. Based on positive ranks.			
b. Based on negative ranks.			

Table 4: Test Statistics Effort

	SRL-HL	FRL-HL	FRL-SRL
Z	-,189[a]	-2,170[a]	-1,261[a]
Asymp. Sig. (2-tailed)	,850	,030	,207
a. Based on negative ranks.			

Table 5: Test Statistics Frustration

	SRL-HL	FRL-HL	FRL-SRL
Z	-,281[a]	-2,149[a]	-1,015[a]
Asymp. Sig. (2-tailed)	,778	,032	,310
a. Based on negative ranks.			

Table 6: Test Statistics Performance

	SRL-HL	FRL-HL	FRL-SRL
Z	-1,107[a]	-1,114[a]	-,120[a]
Asymp. Sig. (2-tailed)	,268	,265	,905
a. Based on positive ranks.			
